# Analysis of the critical dose of radiation therapy in the incidence of Osteoradionecrosis in head and neck cancer patients: a case series

**DOI:** 10.1038/s41405-020-00044-3

**Published:** 2020-09-24

**Authors:** Zain Iqbal, Panayiotis Kyzas

**Affiliations:** 1 The University of Manchester, Manchester, UK; 2 The Department of Oral and Maxillofacial Surgery, North Manchester General Hospital, Northern Care Alliance NHS Group, Manchester, UK

**Keywords:** Oral pathology, Oral cancer

## Abstract

**Introduction:**

Osteoradionecrosis (ORN) is a dramatic complication following radiation therapy (RT) for head and neck tumours. Symptoms include pain, trismus, and malodour. ORN can present with exposed necrotic bone, an orocutaneous fistula, and/ or a pathological fracture.

**Aims:**

To analyse the RT dose responsible for the pathogenesis of ORN and its associated risk factors.

**Methods:**

The data of 17 patients from 2005 to 2017 were retrospectively reviewed from the Pinnacle(3), WebPublication, and Electronic patient records (EPR) provided by Christie Hospital and Pennine Acute NHS Trust.

**Results:**

The mean RT dose that ORN sites received was 57.3 Gy. The mean onset duration for ORN after RT was 640.6 days. six patients (35.2%) developed ORN following post-RT dental extractions.

**Conclusion:**

RT dosages of >57.3 Gy significantly increase the likelihood of developing ORN. Mandibular surgery, post-RT dental extraction, concurrent smoking, and alcohol abuse all amplify the risk of developing ORN.

## Introduction

Osteoradionecrosis (ORN) in the head and neck region is recognised to be a dramatic complication following radiation therapy (RT) for local tumours. ORN is a burden for both clinicians and patients.^1^ ORN describes an irradiated field in which bone undergoes necrosis that eventually becomes exposed through overlying soft tissues.^2^ Most commonly affecting the mandible,^3^ ORN often presents with an array of symptoms and signs that include chronic pain, trismus, malodour, poor wound healing, and in severe cases, orocutaneous fistulae (OCF) and pathological fractures.^1^ Consequentially, the above manifestations of ORN result in a reduced quality of life for patients, which can eventually lead to malnutrition, disfigurement, infection, and dependency on high-dose opiates.^4^ Contributory risk factors for the development of ORN have been widely discussed and well-established in the literature.^1^ Mainly, they point toward both local and systemic factors, which are further discussed in this review. Strategies for the prevention of ORN have been documented and focus on controlling local and systemic risk factors that exist before the start of RT. However, such prevention methods have not yet been protocoled.^2^ Current treatment strategies, despite not being standardised, can give 100% resolution of ORN achieved via conservative and/or radical surgical means.^5^

## Definition

There is controversy in the literature regarding the definition of ORN.^6^ Based on its clinical presentation and observation, ORN can be defined as occurring when ‘irradiated bone becomes devitalised and exposed through the overlying skin or mucosa without healing for 3 months, without recurrence of a tumour’.^7^ Despite this definition being the most frequently used, clinicians argue that it is not complete as it disregards radiological or histological findings consistent with ORN.^6^ Using radiological and histological means to diagnose ORN is also disputed, adding more controversy to the definition.^1^ A minority of patients who develop ORN are seen with radiologically altered bone yet clinically intact mucosa.^7^ It is therefore difficult to define the outlying cases of ORN. Others define ORN as consisting of an area of more than 1cm which has healed for more than six months.^8^ This definition has led to the implementation of the ‘watch and wait’ protocol to fulfil diagnostic criteria.^8^ One could argue that it would be illogical to approach this protocol if early intervention and control of the disease can be delivered.^8^ Nevertheless, an overall consensus has been reached among authors with regards to the presence of devitalised and necrotic bone in their definitions of ORN.^1^

## Classification

**Fig. 1:**
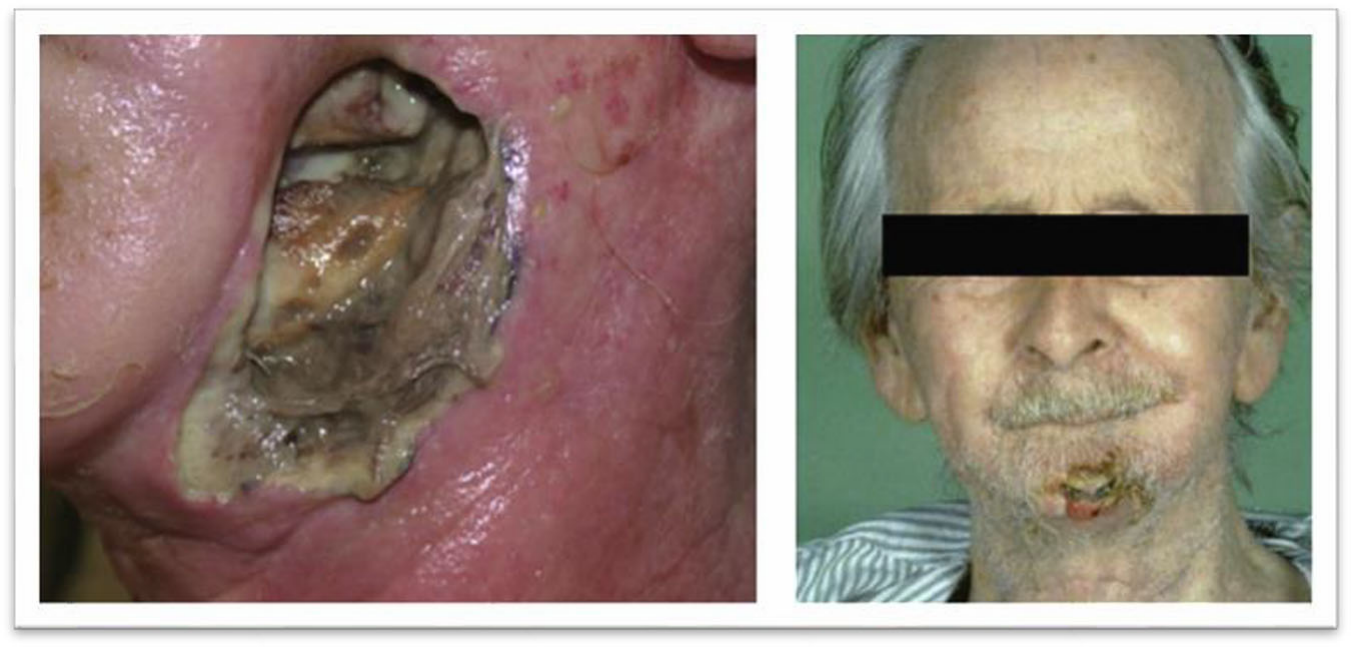
Clinical photographs. These clinical photographs delineate exposed bone in stage III of ORN with orocutaneous fistulae.^1^

Since the first definition of ORN over 30 years ago, the disease has been classified into at least 10 different types.^5^ This is due to the number of definitions and controversies over the years, which have only further proposed newer theories for the pathophysiology of ORN.^1^ In our study, all cases of ORN were graded using the classification developed by Notani et al. This system is widely appreciated and is suitable for mandibular ORN. It quantifies the extent of the ORN lesion into three separate categories based on anatomical boundaries.^9^ Stage I: ORN is confined to the alveolar bone. Stage II: ORN is limited to alveolar bone above the level of the infra-alveolar canal of the mandible. Stage III: ORN extends below the level of the infra-alveolar canal, with an OCF and/or a pathological fracture (Fig. 1). This classification needs concurrent radiographic and clinical evidence in order to differentiate between each stage.^4^ The Notani classification was employed for this study as it was straightforward and easy to use and categorises the extent of bone involvement.^9^ The Notani classification excludes rare cases when ORN that is radiologically confined presents with intact mucosa.

## Pathophysiology

Newer theories of the pathogenesis of ORN have been established, adding to the portfolio of theories that have been proposed for the last 80 years. These theories still influence the ongoing development of medical treatment options.^10^ Marx proposed that tissues, following RT, undergo the process of hypocellularisation. RT leads to cellular death of both soft and hard tissues from direct radiation damage, as well as hypovasularisation of arterioles and arteries due to endarteritis obliterans. Gradual tissue ischaemia and hypoxia consequently give rise to a reduction of the reparative and synthetic capacity of irradiated tissues, eventually producing a non-healing wound.^8^

Endothelial cells are directly, and indirectly, damaged by radiation, and subsequent generation of ROS, respectively. Radiation causes a cytokine cascade which promotes an acute inflammatory response that produces ROS, increasing endothelial injury. Eventual loss of tissue occurs with microvascular necrosis and local ischaemia. A net result of vascular thrombosis from endothelial destruction alters the anatomy of endothelial cell lining permits the entrance of multiple cytokines and subsequent evolution of myofibroblasts from fibroblasts.^11^ The above process is seen in the inferior alveolar artery of the mandible, a common location for ORN. Fibrotic tissue obliterates the artery, resulting in necrosis of the bone and further inflammatory assault. In ORN of the mandible, the compensatory collateral blood supply of the facial artery does not meet the metabolic and oxygen demands of the mandible. As a result, ischaemia and necrosis to the areas supplied by the inferior alveolar artery are inevitable.^12^

In summary, irradiated tissues are left insufficiently perfused, fibrotic, fragile, and hypocellular. This increases the risk of developing ORN if trauma-induced inflammation occurs.^6^

## Risk factors and incidence

Risk factors that predispose patients to ORN are multifactorial and can differ with regards to severity. Despite a lack of clinical studies, risk factors have been implicated and identified in the literature. These risk factors can either be systemic or local. The former is associated with the development of oral cancers and can include malnutrition, immunodeficiency, chronic alcohol abuse, and a long-term smoking history.^13^ The latter refers to events that occur intra-orally, such as the site and size of the tumour, RT site and dosage, poor oral hygiene and dentition, radiation-induced xerostomia, and periodontal disease.^1^

A strong risk factor for the development of ORN is dental-alveolar surgery post-RT, namely, dental extractions and implantations. Around 5% of ORN cases are trauma-induced from dental extractions.^14–16^ For the treatment of tumours near to or involving the mandible, mandibular surgery is another predisposing risk factor for ORN as tissues may struggle to heal post-RT.^17^

A well-recognised risk factor for the occurrence of ORN is dose of RT. Studies have shown that the higher the dose of RT, the higher the risk of ORN.^18^ RT doses to the mandible ranging between 60 and 70 Gy have been heavily associated with the incidence of ORN, while ORN sites that received doses of 50–60 Gy are fewer in number.^19^ With hyper-fractionated RT doses > 70 Gy, reports have shown that incidences of ORN are lower than those of the abovementioned doses.^1^

## Management

### Prevention strategies

Identifying the risk factors that dispose patients to ORN is the first step in devising prevention techniques. Patients undergoing RT treatment for head and neck tumours are advised to have a dental evaluation to identify if any teeth (that may develop caries from RT) need to be extracted.^20^ Adequate healing time for bone and soft tissue must be promoted as failing to do so will increase the likelihood of ORN after commencing RT.^20^ Advising patients to stop smoking and reduce alcohol consumption is of the utmost importance as these risk factors are well-known in contributing to periodontal and dental disease. Following RT treatment, patients should have a postoperative and post-RT dental follow-up appointment arranged where they can receive advice on good oral hygiene.^20^

### Medical

The fibro-atrophic theory has served to provide avenues for newer medical management approaches to ORN.^10^ Tocopherol (vitamin E) serves to minimise the production and regeneration of ROS by protecting cell membranes from oxidative stress due to lipid breakdown. Tocopherol works remarkably well with pentoxifylline, in promoting collagen synthesis and reducing fibrosis.^21^ It works by vasodilating vessel reperfusion tissues and inhibits TNFα production.^10^

### Surgical

Surgery for ORN is usually reserved for Notani stage III cases which may present with pathological fractures of the mandible or an orocutaneous fistula.^21^ For these patients, restoring an adequate blood supply with resection of non-vital bone and a free bony tissue transfer may be the only plausible treatment option.^21^ With regards to management of advanced ORN of the mandible, the most preferred treatment is an osteocutaneous fibula free flap reconstruction.^22^ In a systematic review of 15 articles, the fibula free flap was delineated as the ‘workhorse’ flap for mandibular ORN as it has a high success rate (95.3%).^23^ For less advanced cases of ORN (stages I and II), a debridement of necrotic tissues and/or a sequestrectomy is performed until bleeding bone is visible.^1^

## Patients and methods

Our study included 17 patients who underwent intensity modulated RT (IMRT) for head and neck cancer from 2005 to 2017. Non-Radiotherapy data was critically reviewed and collected from the Electronic patient record (EPR) and Evolve system provided by the Pennine Acute NHS Trust. The following information for each patient was extracted: demographic data, primary/secondary tumour site and size, treatment for primary/secondary tumour, site of ORN, stage of ORN, treatment for ORN, number of days from the completion of RT to diagnosis of ORN, social history risk factors, dental events, and concomitant chemotherapy.

ORN sites were determined from radiographic findings from MRI and CT scans, which were primarily used to rule out recurrent or second primary malignancies. In combination with this, orthopantomograms and clinical descriptions were used to locate the site and stage the severity of the ORN (Table 1).

**Table 1:** Number of cases as per notani stage.

Notani stage	Number of cases
I	5
II	0
III (total)	12
III + OCF	5
III + Pathological fracture	2

RT prescriptions doses for every patient were gathered from a database on the *Pinnacle(3)* and WebPublication systems at The Christie Hospital in Withington, UK. The maximum and mean RT volumetric doses received at specific ORN sites were calculated on the *Pinnacle(3)* system through interpolation of RT dosimetry contours at multiple CT scan planes of ORN lesions.

In cases where ORN developed in the mandible, RT dosimetry doses were calculated at specific sites clearly depicted in patient’s clinical, radiological, and operative reports. Dosimetry data were calculated using the *Pinnacle(3)* system. Radiotherapy treatment courses for our patients involved targeting IMRT at specific tumour sites identified on CT scans, this is known as image guided RT. We computed the mean doses that were targeted at subsequent ORN sites for all subjects involved in this study including cases that did not concern the mandible.

The prescription dose range for the patients was between 52.5 and 66 Gy (Table 2). A total of three patients were prescribed 60 Gy for 30 fractionations. Five patients had a prescription of 63 Gy for 30 fractionations. One patient had 30 fractionations of 66 Gy, and another patient had 52.5 Gy for 20 fractionations.

**Table 2:** Number of ORN cases occurring from corresponding RT doses.

RT mean dose (Gy)	Number of ORN cases
<50	2
<50–60>	13
>60	2

## Results

A summary of all the cases of ORN is represented in Table 3 below. Seventeen cases of ORN were identified in this study, 11 of whom underwent a postoperative RT course. The number of days between the completion of RT and the diagnosis of ORN ranged from 16 to 2304 days (mean: 640.6 days). Our study included nine males and eight females. Patient age range was 50–75 years (mean: 63 years).

**Table 3:** Patient data.

	G/Age	Tumour (site, stage)	ORN site	ORN stage	RT (PN, fractions, DMean)	RT Postop:	Surgery to mandible	Dental operation	CRT to ORN (days)	Smoking (PY)	Alcohol (U/w)
1	M/61	L Retromolar trigone, T4N2 L Infratemporal fossa, T4N0	L Mandibular Condyle	III	60 Gy—30, 56.7 Gy, 63 Gy—30, 47.3 Gy,	Yes	L rim resection, Scapular FF	None	57	65	60
2	F/64	R retromolar trigone, T2N2	R Mandible	I	63 Gy—30, 62.5 Gy	Yes	R rim resection, Scapular FF	None	219	60	60
3	M/76	R Maxilla upper buccal sulcus, T1N1, R Infratemporal fossa, T4N0	R Anterior Maxilla, R External Auditory Meatus	I, I	52.5 Gy—20, 49.9 Gy, 60 Gy—30, 56.7 Gy	Yes	None	None	68, 139	0	5
4	M/61	L Maxilla, T4a	L Zygoma	III	63 Gy—30, 62.8 Gy	Yes	L Coronoidectomy	None	16	120	80
5	F/65	L Floor of Mouth, T4a	L Mandible	III	63 Gy—30, 59.8 Gy	Yes	L anteriorsegmental mandibulectomy	L Sulcoplasty, LL7+8, UL 7 Post- RT Extractions	391	0	20
6	F/70	R Soft Palate, T4a	R Mandible	I	63 Gy—30, 59.8 Gy	Yes	Lip splitmandibulotomy	None	118	140	60
7	F/52	L Tongue Base, T4N2c, Residual	L Mandible	I	66 Gy—30, 59.8 Gy	No	None	None	432	0	40
8	M/68	R Anterior Floor of Mouth, T4N1	R Mandible	III	60 Gy—30, 59.9 Gy	Yes	R segmental mandibulectomy	None	289	100	80
9	F/55	L Buccal, T4N1	L Submental Mandible	III	60 Gy—30, 57.9 Gy	Yes	L hemi-mandibulectomy	Poor oral LL8, UL 4,7+8 Pre-RT Extraction	102	0	50
10	M/57	L Tonsillar,	L Posterior Mandible	III	60 Gy—30, 57.9 Gy	Yes	None	None	2253	60	120
11	M/54	L Tonsillar, T2N0	L Mandible Body and Angle	III	62.5 Gy—25, 53.3 Gy	No	None	2 Stage Lower Clearing, Post-RT Extractions	1689	60	60
12	M/64	L Mandible, T4N0	L Zygoma	III	NR	Yes	L segmental mandibulectomy	None	56	0	20
13	M/50	R Tonsillar, T4N0	R Angle of Mandible	III	65 Gy—30, 57.5 Gy	No	None	LR7 Post- RT Extraction	1944	60	50
14	F/74	R Mandibular, T4N2	R Mandible, L Mandible	III	NR	Yes	R segmental mandibulectomy	None	NR	40	40
15	F/75	R Floor of Mouth, R Tonsillar, T2N2	R Angle of Mandible	III	60 Gy—30, 54.4 Gy	No	R segmental mandibulectomy	LR7 + LL7 Post- RT Extraction	1998	100	120
16	M/67	L Tongue T1N0, R Lateral Tongue, T2N0	R Body of Mandible	III	55 Gy—20, 52.7 Gy	No	None	LR5 + UR5 Post-RT Extraction	2304	30	60
17	F/61	R Tonsillar, L Tonsillar, T2N0	L Body of Mandible	III	60 Gy—30, 57.2 Gy	Yes	Lip splitmandibulotomy	LR7, LR6, Post- RT Extraction LL6+7 Post ORN Dx	120	0	0

*CRT* completion of radiotherapy, *DMean* mean dose, *DX* diagnosis, *FF* free flap, *G* Gender, *Gy* Gray, *L* Left, *R* right, *NR* not retrievable, *PN* prescription, *PY* pack year, *U*/*w* units per week.

Of the 17 patients in the database, 1 patient had developed ORN on 2 separate occasions following 2 separate RT treatment courses. One patient had two co-existing ORN lesions. Overall, there were 19 separate incidences of ORN in these 17 patients.

### Social data

With regards to social history, 12 patients had been simultaneously smoking and consuming alcohol. In total, at the time of diagnosis, 11 patients smoked, and 15 patient consumed alcohol above the recommended weekly unit intake. The smoking pack year for patients ranged from 0 to 140 (median: 60; mean: 49.1) and weekly units ranged from 0 to 120 (median: 60; mean: 54.4).

### Dental data

Upon dental evaluation, ten subjects were found to have poor oral hygiene. However only one developed ORN after undergoing dental extraction before radiotherapy. Six patients suffered ORN after post-RT dental extraction. In two patients, ORN was caused by poor oral health; whether this was due to RT or lack of personal care is unclear. Trauma-induced ORN via post-RT dental extraction occurred in 6 out of 17 patients. In eight ORN cases, the pathogenesis was spontaneous. Only one instance of ORN developed when dental extractions were performed pre-RT.

### Radiotherapy data

From the RT dosimetry data gathered from fifteen patients (Table 2), two cases of ORN occurred after receiving a mean dose (DMean) of <50 Gy. Thirteen cases of ORN occurred with a dose between <50 and 60 Gy>, and two cases with a DMean of >60 Gy. Mean doses ranged from 48.7 to 62.8 Gy (mean: 57.3 Gy), with many incidences resulting from a DMean of >55 Gy. From the data, there is no clear correlation suggesting that an increased DMean increases the severity of ORN. The most common location for ORN in this database was the mandible, recorded for fifteen patients. Twelve patients received postoperative RT, one patient received preoperative RT, and four patients received only RT for the management of their tumours. Interestingly, from the 17 cases of ORN, eight patients received chemoradiotherapy.

### Surgical data

From these seventeen ORN patients, eleven underwent mandibular surgery for the surgical treatment of their primary/recurring tumour.

## Discussion

The findings of our study suggest that there is a significantly high risk of developing ORN following a mean radiation dose of 57.3 Gy for 30 fractions in an irradiated field. This is in keeping with a previous study by Shaw et al. which found that ORN is reported when the average radiation dose received at ORN sites is 57.4 Gy (range: 28.2–74.6 Gy).^4^

Furthermore, a retrospective study done by Lee et al. analysed the ‘dose-effect’ association for ORN of the mandible and recognised an increase in the incidence of ORN in patients who received a tumour radiation dose of >54 Gy.^18^ Tsai et al. suggested in their study that a reduced risk of ORN may be apparent if a dose of <50 Gy was delivered to the whole mandible.^24^ This suggestion could be further studied in the future. Newer methods of delivering RT, such as IMRT techniques, have given rise to fewer ORN cases compared to traditional two-beam techniques.^25^ It is believed that this difference is due to the ability to contour radiation waves around and away from the mandible and salivary glands. This encourages the lowest total doses to these areas. Sparing of the salivary glands is a measure for the prevention of xerostomia and subsequent ORN.^26,27^

The rate of the incidence of ORN preceding our study was deemed to be between 2 and 22%. This was based on multiple ORN classification and varying treatment regimes. The Hyperbaric Oxygen for the Prevention of Osteoradionecrosis (HOPON) study, a multicentre, randomised control trial measured the benefit of hyperbaric oxygen therapy (HBOT) in preventing the onset of ORN in patients that had dental-alveolar surgery with prior RT. This study saw the incidence rates at 6%, however in our study we have not provided an exact incidence rate figure at our centre but are estimated to be ~10%. The rationales behind varying incidence rates are not clear, though incidence rates may potentially differ from one centre to another if management protocols for head and neck cancers are not identical.^28^ No patients in our study had HBOT as part of their treatment or prevention of ORN.

In our study, 35.2% of patients developed ORN shortly after post-RT dental extractions; this result correlates and is in line with other studies. Thorn et al. report that 44 out of 80 (55%) patients in their study had been referred for the treatment of ORN due to post-RT dental extraction. Clinicians treating cancers of the head and neck must strive to educate and ensure their patients establish and maintain healthy dental habits.^29^

From our study, the average time taken to develop ORN from the completion of RT ranged from 0.52 to 75.4 months (mean = 23.9 months). RT can leave long-term fibrotic changes, and inflammatory assaults can reoccur on trauma. Moreover, tissues lose their replicative and synthetic capability, which is outstripped by tissue breakdown. As a result, ORN can present spontaneously.^6^ As opposed to current and contemporary literature, it is supposed that the risk of ORN lessens after extended periods of time. Curi et al. argued that trauma could cause ORN after 2–5 years. Thorn et al. agreed by stating that ORN may result within 36 months after RT.^25,29^ ORN can sometimes result directly from surgical trauma: our study showed four cases of ORN where there was no surgical intervention for the management of primary malignancies, and three cases where surgical trauma was the direct cause of ORN.

Smoking and alcohol have been profoundly associated with an increased risk of developing oral tumours and ORN. It is understood that mucosal membranes may only partially heal after RT. Mucosal membranes are found to be thin and atrophied following RT. It is therefore important to consider that smoking and alcohol irritate and damage mucosal membranes. Combining these two processes together will increase the likelihood of bone exposure and subsequent ORN.^13,15,22^ Furthermore, it’s vital that clinicians educate their patients about the harmful effects of smoking and consuming alcohol so that the incidences of ORN may decrease. In our study, 11 patients smoked (mean: 75.9 PY) and all but one subject consumed alcohol (mean: 57.8 U/w), predisposing them to severe cases of ORN. Nine subjects from our study developed ORN Notani stage III after simultaneous smoking and alcohol use. Recent studies by Owosho et al. and Tsai et al. found that patients who continue to consume alcohol and smoke cigarettes concurrently are at a threefold greater risk of developing ORN.^5^

Our study found that a key risk factor for the pathogenesis of ORN is surgery involving the mandible for tumour resection. Eight ORN sites of the total 17 cases corresponded to the mandibular resections performed in those surgical sites. The most common surgical procedure associated with ORN in our study was segmental mandibulectomy.

### Limitations of the study

This study was based on 19 cases of ORN that occurred in a total of 17 patients at a single maxillofacial cancer centre over a period of 12 years. Our study was a case series with a small sample of patients, and we acknowledge that there are stronger forms of study designs that could be used for research in this topic to yield more reliable conclusions.

In the future, we suggest prospective studies should be conducted in multiple centres to investigate the risk factors of ORN. A systematic review and meta-analysis discovering mean RT doses responsible for the pathogenesis of ORN in head and neck cancer patients should also be carried out. From this, we hope that the holistic management of head and neck tumours are influenced and that the incidence rates of ORN decline. RT prescriptions and dosimetry findings could not be retrieved for 2 out of 17 patients.

## Conclusion

ORN is a dramatic complication following RT that often requires surgical intervention. A mean radiation dose 57.3 Gy predisposes head and neck patients to developing ORN. Post-RT dental extractions, mandibular surgery, excessive smoking, and alcohol consumption are all major risk factors in the pathogenesis of ORN. To minimise the risk of ORN, a strict dental health regime must be offered for patients receiving a RT dosage >57.3 Gy.
